# Gastric Carcinoma with Osteoclast-Like Giant Cells Coexisting with Gastrointestinal Spindle Cell Tumor

**DOI:** 10.1155/2013/240758

**Published:** 2013-06-19

**Authors:** Christos Poulios, Triantafyllia Koletsa, Antonis Goulas, Georgia Karayannopoulou, Eleni Vrettou, Ioannis Kostopoulos

**Affiliations:** ^1^Department of Pathology, Medical School, Aristotle University of Thessaloniki, 54124 Thessaloniki, Greece; ^2^1st Department of Pharmacology, Medical School, Aristotle University of Thessaloniki, 54124 Thessaloniki, Greece

## Abstract

Reactive multinucleated osteoclast-like giant cells (OGCs) have been described in a variety of neoplasms but rarely in gastric carcinomas. Reported herein is a case of an 81-year-old Caucasian male presented with upper abdominal pain and dysphagia. Esophagogastroscopy revealed an ulcerative mass and a specimen of subtotal gastrectomy and lower esophagectomy was sent for histologic examination. At the gastroesophageal junction an exophytic tumor, measured 2.2 cm in greatest diameter, was observed. Sections from the tumor showed gastric adenocarcinoma, stage pT1bpN0. Diffusely among the neoplastic cells multinucleated giant cells, resembling osteoclasts, were observed, which were positive for CD68, lysozyme, and vimentin and negative for AE1/AE3, CK8/18, hHCG, and LMP1. Moreover, in a random section from the gastric fundus, a spindle cell lesion, sized 0.6 cm, was revealed, which was positive for CD117 and CD34 antigens and was diagnosed as gastrointestinal stromal tumor (GIST). The presence of OGCs is an uncommon finding in gastric carcinomas and by analogy to breast and pancreatic carcinomas it could characterize a rare distinct morphological variant of gastric adenocarcinoma. Due to the limited number of the reported cases, the prognostic value of OGCs is under discussion. Furthermore, pathologists should be aware that incidental GIST may accompany any tumor.

## 1. Introduction

Nonneoplastic multinucleated giant cells resembling osteoclasts are a relatively common finding in neoplasms of various organs, such as skin, thyroid gland, ovary, breast, kidney, prostate and especially pancreatic adenocarcinoma. Undifferentiated carcinoma with osteoclast-like giant cells (OGCs) was first described by Rosai in 1968 [1] and has been classified by WHO in 2000 as a subtype of pancreatic undifferentiated carcinoma [2]. However, OGCs are rarely observed in gastric carcinomas with only 16 cases reported in the literature thus far [3–9]. 

Although adenocarcinoma is the most common type of gastric neoplasms, the synchronous existence of gastrointestinal stromal tumor (GIST) is an uncommon event. GISTs can be detected simultaneously with other malignancies, even if they originate from different cell layers [10]. Herein, the extremely rare occurrence of gastric adenocarcinoma with OGCs and GIST is presented.

## 2. Case Report

An 81-year-old male patient presented with upper abdominal pain and dysphagia. There was no history of melena, hematochezia, or jaundice. Laboratory tests revealed only mild anaemia. During esophagogastroscopy an ulcerative mass in the gastroesophageal junction was clearly observed. Since the mass biopsy showed an adenocarcinoma, surgery was scheduled. A specimen of subtotal gastrectomy and lower esophagectomy was sent for histologic examination.

*Histologic Findings*. On gross examination, an exophytic tumor, located in the gastro-esophageal junction and measured 2.2 cm in greatest diameter, was observed. From the adjacent to the stomach adipose tissue, 13 lymph nodes were isolated.

**Figure 1:**
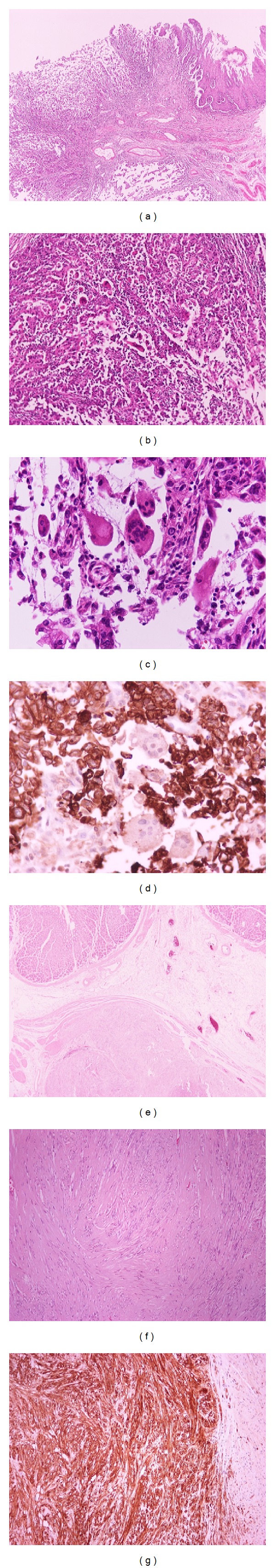
Poorly differentiated adenocarcinoma on gastroesophageal junction (a) with multinucleated giant cells (b), (c), which are negative to keratins AE1AE3 (d). Coincidental GIST (e), (f) positive to CD117/c-kit antigen (g). (a) H-E ×40, (b) H-E ×100, (c) H-E ×400, (d) IHC ×400, (e) H-E ×20, (f) H-E ×400, and (g) IHC ×400.

H-E stained sections from various parts of the tumor showed infiltration of gastro-esophageal mucosa by poorly differentiated adenocarcinoma (Figure 1(a)). There was a high mitotic rate. Diffusely among the neoplastic cells, giant multinuclear cells, resembling osteoclasts, were observed (Figure 1(b)). The OGCs had an eosinophilic cytoplasm and contained 3 to 20 round- or oval-shaped nuclei (Figure 1(c)). The neoplasm infiltrated in depth up to the gastric submucosa and was extended to the esophageal mucosa as well. There was dense inflammatory infiltration at the periphery of the tumor consisting of lymphocytes, plasma cells, and neutrophils. Areas of necrosis and intraluminal abscesses were also observed. None of the isolated lymph nodes showed evidence of metastasis. The surgical resection margins were free of carcinomatous cells. Immunohistochemically, the neoplastic cells were positive for keratins cam5.2 and AE1/AE3 and negative for CD68 and vimentin. On the other hand, OGCs were positive for CD68 antigen, lysozyme, and vimentin and negative for keratins (Figure 1(d)), suggesting a reactive nature. Immunostaining for hHCG, AFP, and LMP1 was negative. 

In a random section taken from the gastric fundus, a nodular spindle cell lesion was revealed within the muscularis propria (Figure 1(e)), measuring 6 mm in the greatest diameter. The cells were predominantly arranged in bundles (Figure 1(f)) and on immunohistochemical analysis displayed positivity to the CD117/c-kit (Figure 1(g)) and CD34 antigens, whereas they were negative for smooth muscle actin (SMA), desmin, caldesmon, and S-100 protein. There were mild atypia and rare mitoses; the Ki67/mib1 mitotic index was positive for less than 3% of the total spindle cells detected. No signs of necrosis were found. All the previous histologic findings were consistent with GIST. The diagnosis of gastric adenocarcinoma with osteoclast-like giant cells coexisting with GIST was set. Postoperative followup showed that the patient has remained free of recurrence the last 12 months. 

## 3. Discussion

Extraskeletal tumors containing OGCs are more often reported in the breast and pancreas, while in stomach there are only 16 reported cases up to date (Table 1). A review of the literature showed that gastric adenocarcinoma with OGCs affects mainly male patients (15 out of a total of 17) with an average age of 71 years, it arises predominantly in the gastric cardia or corpus, and it is apparently characterized by poor differentiation. In all cases, the OGCs were positive for CD68 and negative for keratins (mainly AE1/AE3), indicating their reactive nature. In some of the reported cases the neoplasmatic cells were positive either for EBER *in situ* hybridization or the LMP1 protein, and the authors raised the possibility of a lymphoepithelioma-like carcinoma [6]. The morphology of these giant cells is of foreign body type, Langhans, or osteoclast-like [8]. In any event, immunohistochemistry should always be performed in such cases, in order to differentiate OGCs from multinuclear anaplastic cancer cells, which may have multiple nuclei.

As OGCs are generally considered to represent a host response to the neoplasm, some authors have suggested that their presence in the context of a gastric carcinoma is associated with a more favorable prognosis [5], as initially thought for the undifferentiated pancreatic carcinoma with OGCs and later suggested that it is characterized by a poor prognosis with median survival only 12 months [2]. Recently, WHO has recognized carcinoma with osteoclast-like stroma giant cells as a distinct type of invasive breast carcinoma of no special type, in which prognosis does not appear to be influenced by the presence of stroma giant cells [11]. The presence of OGCs in gastric carcinoma seems to correspond to a different morphological variant of adenocarcinoma and the limited number of the cases cannot allow any definite conclusion about prognosis. 

There are only few reported cases of concurrent existence of gastric adenocarcinoma and GIST, not including a case of adenocarcinoma with OGCs. Globally, GISTs account for the 1% of all gastrointestinal malignancies with the stomach being the most common site. GISTs more often develop in a sporadic fashion and they are usually discovered incidentally, during an operation or a histological examination performed because of nontumorous disease or due to another malignancy [10], as happened in this particular case. Incidental GIST can be found in 35% of stomach-resected patients with gastric cancer [12] and in 10% of esophagogastric resections for esophageal or esophagogastric junction carcinomas [13]. The etiology and the mechanism behind this coexistence, if any, remain unknown. In experimental models it has been demonstrated that the combination of different factors may lead to different neoplasms, such as gastric cancer and leiomyosarcoma [14].

Conclusively, when GISTs are submucosal or subserosal they are difficult to diagnose preoperatively. Surgeons and pathologists should be aware of coincidental GISTs in order to minimize the possibility to be overlooked, especially in cases where additional therapy is warranted. Further studies are needed in order to define if there is any common causative factor for synchronous development of epithelial and nonepithelial tumors in the digestive tract. In addition, although the presence of OGCs is considered to have no prognostic value in pancreatic and breast carcinomas, the limited number of cases in stomach does not allow drawing any reliable conclusion about prognosis, so far. These stroma giant cells could only characterize a rare morphological variant of gastric adenocarcinoma.

## Figures and Tables

**Table 1:** Clinicopathologic characteristics of all reported cases of gastric adenocarcinoma with OGCs.

Case no.	References no.	Sex	Age	Stomach localization	Tumor size (cm)	EBV	Stage (pTNM)
1	[5]	M	63	Lesser curvature	9 × 7	+	T2N1M0
2	[5]	M	53	Cardia	3 × 4	+	T2N2M0
3	[5]	M	61	Cardia	10 × 7	+	T2N1M0
4	[5]	M	76	Lesser curvature	3.5 × 3	+	T2N1M0
5	[3]	F	64	Antrum	3.5 × 1.9	NA	T1N2M0
6	[7]	M	50	Cardia	NA	NA	T3N0M0
7	[4]	F	70	Cardia	10 × 10 × 6	+	T3N2M0
8	[9]	M	78	Antrum	10 × 8	−	T3N2M1
9	[8]	M	100	Lesser curvature	5 × 4	−	T3N0M0
10	[6]	M	69	Corpus	5.5	+	T3N0M0
11	[6]	M	71	Cardia	6	+	T2N1M0
12	[6]	M	64	Corpus	6.3	+	T3N1M0
13	[6]	M	69	Corpus	2.3	+	T1N0M0
14	[6]	M	65	Antrum	10	−	T2N1M0
15	[6]	M	84	Antrum	5	−	T2N1M0
16	[6]	M	86	Corpus	7.2	−	T4N1M0
Present case		M	81	Cardioesophageal junction	2.2	−	T1bN0M0

No: number, Ref: reference, M: male, F: female, NA: not available, +: presence of EBV demonstrated either by immunohistochemistry or in situ hybridization, −: absence of EBV.
